# Nitrato[*N*,*N*,*N*′,*N*′-tetra­kis(1*H*-benzimid­azol-2-ylmeth­yl)ethane-1,2-diamine]­calcium(II) nitrate methanol trisolvate

**DOI:** 10.1107/S1600536808037859

**Published:** 2008-11-26

**Authors:** Bei Huang, Yamei Pei, Li Wang

**Affiliations:** a Key Laboratory of Pesticides and Chemical Biology, Department of Chemistry, Central China Normal University, Wuhan, Hubei 430079, People’s Republic of China

## Abstract

In the title compound, [Ca(NO_3_)(C_34_H_32_N_10_)]NO_3_·3CH_4_O, the Ca^II^ ion is coordinated by six N atoms of the EDTB ligand {EDTB is *N*,*N*,*N*′,*N*′-tetra­kis[(2-benzimidazol­yl)meth­yl]-1,2-ethanediamine} and two O atoms from the nitrate ligand, to form a distorted dodeca­hedral geometry. The crystal packing is stabilized by inter­molecular N—H⋯O, N—H⋯N and O—H⋯O hydrogen bonds, which link the constituent units into a three-dimensional network. The uncoordinated nitrate anion is disordered over two sites, with fixed occupancies of 0.77 and 0.23.

## Related literature

For general background, see: Chen *et al.* (2004 ▶); Liao *et al.* (2001 ▶); Pei *et al.* (2007 ▶). For the synthesis of the EDTB ligand, see: Hendriks *et al.* (1982 ▶).
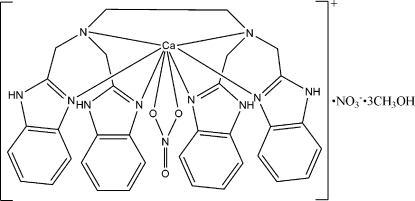


## Experimental

### 

#### Crystal data

[Ca(NO_3_)(C_34_H_32_N_10_)]NO_3_·3CH_4_O
*M* *_r_* = 840.92
Monoclinic, 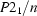

*a* = 9.5945 (4) Å
*b* = 25.3531 (11) Å
*c* = 17.8402 (7) Å
β = 105.599 (1)°
*V* = 4179.8 (3) Å^3^
*Z* = 4
Mo *K*α radiation
μ = 0.22 mm^−1^
*T* = 294 (2) K
0.20 × 0.20 × 0.10 mm

#### Data collection

Bruker SMART CCD area-detector diffractometer
Absorption correction: multi-scan (*SADABS*; Sheldrick, 2001 ▶) *T*_min_ = 0.948, *T*_max_ = 0.979
43547 measured reflections
8189 independent reflections
4825 reflections with *I* > 2σ(*I*)
*R*_int_ = 0.146

#### Refinement

*R*[*F* ^2^ > 2σ(*F* ^2^)] = 0.065
*wR*(*F* ^2^) = 0.171
*S* = 0.90
8189 reflections
575 parameters
23 restraints
H-atom parameters constrained
Δρ_max_ = 0.47 e Å^−3^
Δρ_min_ = −0.38 e Å^−3^

### 

Data collection: *SMART* (Bruker, 2001 ▶); cell refinement: *SAINT-Plus* (Bruker, 2001 ▶); data reduction: *SAINT-Plus*; program(s) used to solve structure: *SHELXS97* (Sheldrick, 2008 ▶); program(s) used to refine structure: *SHELXL97* (Sheldrick, 2008 ▶); molecular graphics: *SHELXTL* (Sheldrick, 2008 ▶); software used to prepare material for publication: *SHELXTL*.

## Supplementary Material

Crystal structure: contains datablocks global, I. DOI: 10.1107/S1600536808037859/ci2674sup1.cif

Structure factors: contains datablocks I. DOI: 10.1107/S1600536808037859/ci2674Isup2.hkl

Additional supplementary materials:  crystallographic information; 3D view; checkCIF report

## Figures and Tables

**Table 1:** Selected bond lengths (Å)

Ca1—O1	2.440 (2)
Ca1—N9	2.470 (2)
Ca1—N5	2.477 (2)
Ca1—N3	2.521 (2)
Ca1—N7	2.543 (2)
Ca1—O2	2.641 (2)
Ca1—N1	2.647 (2)
Ca1—N2	2.649 (2)

**Table 2:** Hydrogen-bond geometry (Å, °)

*D*—H⋯*A*	*D*—H	H⋯*A*	*D*⋯*A*	*D*—H⋯*A*
N4—H4*A*⋯O5^i^	0.86	2.46	3.208 (8)	145
N4—H4*A*⋯O6^i^	0.86	2.09	2.888 (10)	154
N6—H6*A*⋯O2^ii^	0.86	2.26	3.099 (3)	165
N6—H6*A*⋯O3^ii^	0.86	2.45	3.118 (3)	135
N10—H10⋯O1^iii^	0.86	1.94	2.789 (3)	172
O7—H7*A*⋯O4^iv^	0.82	2.39	3.109 (11)	147
N8—H8⋯O4	0.86	1.92	2.730 (7)	157
O8—H8*A*⋯O9	0.82	1.86	2.655 (6)	164
O9—H9⋯O5	0.82	2.14	2.929 (9)	160
C9—H9*B*⋯O7	0.97	2.39	3.274 (5)	151
C17—H17*A*⋯O8	0.97	2.33	3.290 (5)	168
C19—H19*B*⋯O8	0.97	2.45	3.378 (5)	160

Symmetry codes: (i) 
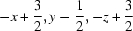
; (ii) 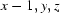; (iii) 
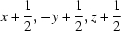
; (iv) 
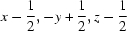
.

**Fig. 1.**
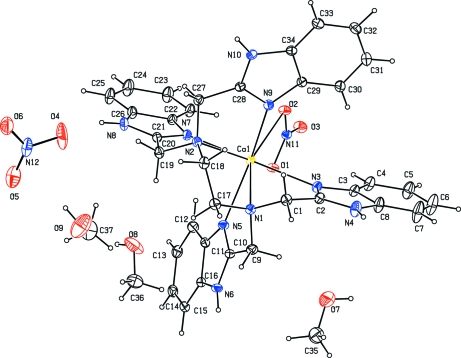
The asymmetric unit of the title compound. Displacement ellipsoids are drawn at the 50% probability level. Only the major component of the disordered nitrate ion is shown.

**Fig. 2.**
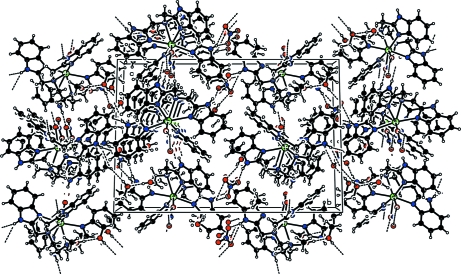
The crystal structure of the title compound, viewed along the a axis. Hydrogen bonds are shown as dashed lines.

## supplementary crystallographic information

### Comment

Benzimidazole containing ligands and their metal complexes have been reported (Chen *et al.*, 2004; Liao *et al.*, 2001; Pei *et al.*, 2007). In continuation of our work on these types of ligands, we report here the crystal structure of the title compound, [Ca(EDTB)(NO_3_)]^+^.NO_3_^-^.3CH_3_OH (EDTB is *N*,*N*,*N*',*N*'-Tetrakis-[(2-benzimidazolyl)methyl] -1,2-ethanediamine).

The asymmetric unit consists of one [Ca(EDTB)(NO_3_)]^+^ cationic unit, one uncoordinated nitrate anion and three methanol solvent molecules (Fig. 1). The structure of the monocation shows an eight-coordinated geometry with Ca^II^ ion at the centre of a distorted dodecahedral environment (Table 1). The Ca^II^ ion is coordinated by four benzimidazole (bzim) N atoms (N3,N5,N7 and N9) and two amine N atoms (N1 and N2) from the EDTB ligand, and two O atoms (O1/O2) from the coordinated nitrate ion.

The coordination cations, uncoordinated nitrate anions and free methanol molecules are interlinked into a three-dimensional network by a combination of six N—H···O, two O—H···O and three C—H···O hydrogen bonds (Table 2 and Fig. 2).

### Experimental

*N*,*N*,*N*',*N*'-Tetrakis-[(2-benzimidazolyl)methyl] -1,2-ethanediamine (EDTB) ligand was synthesized according to the literature method (Hendriks *et al.*, 1982). EDTB (0.58 g, 1.0 mmol) in hot methanol (20 ml) was added slowly to a solution of Ca(NO_3_)_2_.4H_2_O (0.236 g, 1.0 mmol) in methanol (10 ml) and the mixture was stirred for 1 h. After filtration, the solution was allowed to stand at room temperature. Block-shaped pale red crystals suitable for X-ray analysis were obtained after several days in 45% yield.

### Refinement

The nitrate anion is disordered over two sites. The site occupancies were initially refined to 0.77 (1) and 0.23 (1) for the major and minor components, respectively, and later fixed. In both disorder components, the N—O distances were restrained to 1.38 (1) Å and the O···O distances were restrained to be equal. C-bound H atoms were positioned geometrically (C—H = 0.93–0.97 Å) and were refined as riding with *U*_iso_(H) = 1.2–1.5*U*_eq_(C). O– and N-bound H atoms were first located in a difference map and then constrained to be at their ideal positions (O—H = 0.82 Å and N—H = 0.86 Å) with *U*_iso_ values set at 1.2*U*_eq_(N) and 1.5*U*_eq_(O).

### Crystal data

**Table:**

[Ca(NO_3_)(C_34_H_32_N_10_)]NO_3_·3CH_4_O	*F*_000_ = 1768
*M**_r_* = 840.92	*D*_x_ = 1.336 Mg m^−^^3^
Monoclinic, *P*2_1_/*n*	Mo *K*α radiation λ = 0.71073 Å
Hall symbol: -P 2yn	Cell parameters from 6962 reflections
*a* = 9.5945 (4) Å	θ = 2.2–21.4º
*b* = 25.3531 (11) Å	µ = 0.22 mm^−^^1^
*c* = 17.8402 (7) Å	*T* = 294 (2) K
β = 105.599 (1)º	Block, pale-red
*V* = 4179.8 (3) Å^3^	0.20 × 0.20 × 0.10 mm
*Z* = 4	

### Data collection

**Table:**

Bruker SMART CCD area-detector diffractometer	8189 independent reflections
Radiation source: fine-focus sealed tube	4825 reflections with *I* > 2σ(*I*)
Monochromator: graphite	*R*_int_ = 0.146
*T* = 294(2) K	θ_max_ = 26.0º
φ and ω scans	θ_min_ = 1.4º
Absorption correction: multi-scan(SADABS; Sheldrick, 2001)	*h* = −11→11
*T*_min_ = 0.948, *T*_max_ = 0.979	*k* = −31→31
43547 measured reflections	*l* = −22→22

### Refinement

**Table:**

Refinement on *F*^2^	Secondary atom site location: difference Fourier map
Least-squares matrix: full	Hydrogen site location: inferred from neighbouring sites
*R*[*F*^2^ > 2σ(*F*^2^)] = 0.065	H-atom parameters constrained
*wR*(*F*^2^) = 0.171	*w* = 1/[σ^2^(*F*_o_^2^) + (0.0978*P*)^2^] where *P* = (*F*_o_^2^ + 2*F*_c_^2^)/3
*S* = 0.90	(Δ/σ)_max_ = 0.001
8189 reflections	Δρ_max_ = 0.47 e Å^−^^3^
575 parameters	Δρ_min_ = −0.37 e Å^−^^3^
23 restraints	Extinction correction: none
Primary atom site location: structure-invariant direct methods	

### Special details

**Table:**

Geometry. All e.s.d.'s (except the e.s.d. in the dihedral angle between two l.s. planes) are estimated using the full covariance matrix. The cell e.s.d.'s are taken into account individually in the estimation of e.s.d.'s in distances, angles and torsion angles; correlations between e.s.d.'s in cell parameters are only used when they are defined by crystal symmetry. An approximate (isotropic) treatment of cell e.s.d.'s is used for estimating e.s.d.'s involving l.s. planes.
Refinement. Refinement of *F*^2^ against ALL reflections. The weighted *R*-factor *wR* and goodness of fit *S* are based on *F*^2^, conventional *R*-factors *R* are based on *F*, with *F* set to zero for negative *F*^2^. The threshold expression of *F*^2^ > σ(*F*^2^) is used only for calculating *R*-factors(gt) *etc*. and is not relevant to the choice of reflections for refinement. *R*-factors based on *F*^2^ are statistically about twice as large as those based on *F*, and *R*- factors based on ALL data will be even larger.

### Fractional atomic coordinates and isotropic or equivalent isotropic displacement parameters (Å^2^)

**Table:**

	*x*	*y*	*z*	*U*_iso_*/*U*_eq_	Occ. (<1)
Ca1	0.93863 (6)	0.24863 (2)	0.58359 (3)	0.03938 (18)	
C1	0.7836 (3)	0.14868 (11)	0.65167 (19)	0.0572 (8)	
H1A	0.7070	0.1277	0.6628	0.069*	
H1B	0.8678	0.1467	0.6962	0.069*	
C2	0.8201 (3)	0.12762 (11)	0.58169 (19)	0.0540 (8)	
C3	0.8889 (3)	0.12150 (12)	0.47646 (19)	0.0588 (8)	
C4	0.9379 (4)	0.12879 (15)	0.4103 (2)	0.0768 (11)	
H4	0.9689	0.1618	0.3986	0.092*	
C5	0.9393 (5)	0.08604 (18)	0.3627 (3)	0.1052 (15)	
H5	0.9736	0.0900	0.3190	0.126*	
C6	0.8897 (7)	0.03698 (19)	0.3796 (3)	0.136 (2)	
H6	0.8907	0.0088	0.3464	0.163*	
C7	0.8402 (7)	0.02916 (18)	0.4427 (3)	0.138 (2)	
H7	0.8071	−0.0037	0.4534	0.165*	
C8	0.8404 (5)	0.07194 (13)	0.4911 (2)	0.0808 (11)	
C9	0.5963 (3)	0.20632 (12)	0.57942 (19)	0.0571 (8)	
H9A	0.5191	0.2042	0.6049	0.069*	
H9B	0.5873	0.1765	0.5444	0.069*	
C10	0.5819 (3)	0.25616 (11)	0.53395 (17)	0.0459 (7)	
C11	0.6306 (3)	0.32351 (11)	0.47183 (16)	0.0460 (7)	
C12	0.6945 (4)	0.36285 (12)	0.43761 (19)	0.0595 (8)	
H12	0.7945	0.3664	0.4495	0.071*	
C13	0.6052 (4)	0.39592 (12)	0.38618 (19)	0.0643 (9)	
H13	0.6457	0.4223	0.3626	0.077*	
C14	0.4564 (4)	0.39146 (12)	0.3680 (2)	0.0650 (9)	
H14	0.3989	0.4148	0.3326	0.078*	
C15	0.3923 (3)	0.35281 (12)	0.40159 (19)	0.0594 (8)	
H15	0.2922	0.3498	0.3902	0.071*	
C16	0.4812 (3)	0.31902 (11)	0.45229 (17)	0.0475 (7)	
C17	0.7243 (3)	0.23028 (13)	0.71045 (18)	0.0564 (8)	
H17A	0.6663	0.2620	0.6968	0.068*	
H17B	0.6743	0.2069	0.7376	0.068*	
C18	0.8664 (3)	0.24460 (12)	0.76340 (18)	0.0558 (8)	
H18A	0.8508	0.2620	0.8089	0.067*	
H18B	0.9208	0.2126	0.7808	0.067*	
C19	0.8988 (3)	0.33411 (11)	0.72146 (19)	0.0562 (8)	
H19A	0.9211	0.3500	0.7728	0.067*	
H19B	0.7945	0.3343	0.7000	0.067*	
C20	0.9683 (3)	0.36492 (11)	0.67018 (17)	0.0490 (7)	
C21	1.0657 (3)	0.38720 (11)	0.57887 (18)	0.0492 (7)	
C22	1.1232 (4)	0.39029 (12)	0.5158 (2)	0.0658 (9)	
H22	1.1240	0.3608	0.4848	0.079*	
C23	1.1795 (4)	0.43753 (13)	0.4995 (2)	0.0844 (12)	
H23	1.2177	0.4404	0.4569	0.101*	
C24	1.1788 (5)	0.48114 (14)	0.5472 (3)	0.0960 (14)	
H24	1.2177	0.5127	0.5355	0.115*	
C25	1.1242 (5)	0.47959 (14)	0.6094 (3)	0.0881 (12)	
H25	1.1257	0.5091	0.6406	0.106*	
C26	1.0661 (3)	0.43219 (11)	0.6248 (2)	0.0599 (8)	
C27	1.1042 (3)	0.27818 (12)	0.77325 (17)	0.0532 (8)	
H27A	1.1090	0.2774	0.8282	0.064*	
H27B	1.1536	0.3097	0.7632	0.064*	
C28	1.1768 (3)	0.23033 (11)	0.75227 (16)	0.0471 (7)	
C29	1.2351 (3)	0.16532 (11)	0.68917 (17)	0.0499 (7)	
C30	1.2468 (4)	0.12775 (13)	0.6351 (2)	0.0702 (10)	
H30	1.1819	0.1267	0.5859	0.084*	
C31	1.3574 (4)	0.09212 (15)	0.6566 (2)	0.0813 (11)	
H31	1.3682	0.0666	0.6212	0.098*	
C32	1.4533 (4)	0.09323 (14)	0.7297 (2)	0.0750 (10)	
H32	1.5260	0.0679	0.7428	0.090*	
C33	1.4440 (4)	0.13041 (14)	0.7828 (2)	0.0664 (9)	
H33	1.5094	0.1314	0.8319	0.080*	
C34	1.3342 (3)	0.16646 (11)	0.76108 (17)	0.0494 (7)	
C35	0.3284 (6)	0.1315 (2)	0.4261 (3)	0.1300 (19)	
H35A	0.3148	0.1053	0.4623	0.195*	
H35B	0.2691	0.1232	0.3750	0.195*	
H35C	0.3015	0.1655	0.4415	0.195*	
C36	0.4021 (5)	0.3258 (2)	0.6834 (4)	0.136 (2)	
H36A	0.3823	0.3240	0.7334	0.204*	
H36B	0.3868	0.2917	0.6591	0.204*	
H36C	0.3385	0.3510	0.6513	0.204*	
C37	0.6019 (7)	0.4540 (3)	0.5955 (4)	0.147 (2)	
H37A	0.5568	0.4879	0.5834	0.220*	
H37B	0.5512	0.4285	0.5584	0.220*	
H37C	0.7008	0.4561	0.5936	0.220*	
N1	0.7361 (2)	0.20398 (9)	0.63783 (14)	0.0480 (6)	
N2	0.9530 (2)	0.27953 (9)	0.72732 (13)	0.0463 (6)	
N3	0.8747 (3)	0.15622 (9)	0.53404 (14)	0.0510 (6)	
N4	0.7990 (4)	0.07761 (10)	0.55867 (19)	0.0832 (9)	
H4A	0.7653	0.0530	0.5821	0.100*	
N5	0.6927 (2)	0.28340 (9)	0.52322 (14)	0.0488 (6)	
N6	0.4545 (2)	0.27564 (10)	0.49387 (15)	0.0530 (6)	
H6A	0.3708	0.2635	0.4938	0.064*	
N7	1.0047 (2)	0.34531 (9)	0.60981 (14)	0.0480 (6)	
N8	1.0045 (3)	0.41587 (10)	0.68268 (16)	0.0637 (7)	
H8	0.9917	0.4350	0.7201	0.076*	
N9	1.1344 (2)	0.20637 (9)	0.68459 (13)	0.0507 (6)	
N10	1.2934 (3)	0.20879 (10)	0.79974 (14)	0.0539 (6)	
H10	1.3361	0.2192	0.8461	0.065*	
N11	1.0825 (3)	0.25741 (8)	0.45680 (15)	0.0452 (6)	
O1	0.9506 (2)	0.26521 (8)	0.45075 (11)	0.0521 (5)	
O2	1.1601 (2)	0.24507 (8)	0.52220 (12)	0.0590 (6)	
O3	1.1313 (2)	0.26311 (10)	0.40031 (13)	0.0689 (6)	
N12	0.8396 (12)	0.4913 (3)	0.8304 (5)	0.109 (5)	0.77
O4	0.9043 (11)	0.4561 (3)	0.8003 (5)	0.205 (5)	0.77
O5	0.7299 (9)	0.5142 (3)	0.7906 (5)	0.155 (4)	0.77
O6	0.8972 (10)	0.5031 (4)	0.9014 (5)	0.142 (4)	0.77
O7	0.4716 (5)	0.13242 (19)	0.4254 (3)	0.1470 (14)	
H7A	0.4895	0.1063	0.4025	0.220*	
O8	0.5364 (4)	0.34029 (15)	0.6924 (4)	0.177 (2)	
H8A	0.5386	0.3713	0.6795	0.265*	
O9	0.5977 (8)	0.4396 (2)	0.6665 (3)	0.208 (2)	
H9	0.6531	0.4582	0.6988	0.312*	
O5'	0.7826 (15)	0.5023 (8)	0.7646 (13)	0.128 (10)	0.23
O6'	0.9780 (19)	0.4577 (8)	0.8264 (11)	0.101 (6)	0.23
N12'	0.877 (2)	0.4910 (9)	0.8299 (11)	0.13 (2)	0.23
O4'	0.834 (3)	0.4922 (11)	0.8925 (13)	0.118 (9)	0.23

### Atomic displacement parameters (Å^2^)

**Table:**

	*U*^11^	*U*^22^	*U*^33^	*U*^12^	*U*^13^	*U*^23^
Ca1	0.0386 (3)	0.0402 (3)	0.0392 (3)	−0.0034 (2)	0.0104 (2)	0.0009 (2)
C1	0.065 (2)	0.0470 (17)	0.065 (2)	−0.0038 (14)	0.0252 (16)	0.0093 (15)
C2	0.0602 (19)	0.0429 (16)	0.0598 (19)	−0.0062 (14)	0.0177 (16)	0.0041 (14)
C3	0.069 (2)	0.0490 (18)	0.059 (2)	−0.0120 (15)	0.0190 (17)	−0.0078 (15)
C4	0.102 (3)	0.065 (2)	0.072 (2)	−0.0244 (19)	0.038 (2)	−0.0197 (18)
C5	0.152 (4)	0.090 (3)	0.092 (3)	−0.031 (3)	0.064 (3)	−0.033 (3)
C6	0.223 (6)	0.078 (3)	0.131 (4)	−0.047 (4)	0.092 (5)	−0.048 (3)
C7	0.236 (7)	0.066 (3)	0.147 (5)	−0.056 (3)	0.113 (5)	−0.039 (3)
C8	0.120 (3)	0.049 (2)	0.085 (3)	−0.0203 (19)	0.050 (2)	−0.0139 (18)
C9	0.0488 (18)	0.0552 (18)	0.070 (2)	−0.0111 (14)	0.0204 (16)	0.0041 (16)
C10	0.0356 (15)	0.0493 (17)	0.0527 (17)	−0.0026 (13)	0.0117 (13)	−0.0022 (13)
C11	0.0450 (17)	0.0437 (16)	0.0505 (17)	0.0017 (12)	0.0150 (13)	−0.0027 (13)
C12	0.0568 (19)	0.0535 (18)	0.071 (2)	−0.0020 (15)	0.0220 (17)	0.0052 (16)
C13	0.078 (2)	0.0466 (18)	0.068 (2)	−0.0015 (16)	0.0188 (19)	0.0037 (15)
C14	0.074 (2)	0.0493 (19)	0.065 (2)	0.0160 (17)	0.0062 (18)	−0.0016 (16)
C15	0.0529 (18)	0.0546 (19)	0.066 (2)	0.0103 (15)	0.0071 (16)	−0.0084 (16)
C16	0.0443 (17)	0.0490 (17)	0.0481 (17)	0.0025 (13)	0.0107 (14)	−0.0079 (13)
C17	0.060 (2)	0.0584 (18)	0.060 (2)	−0.0037 (15)	0.0324 (17)	0.0069 (15)
C18	0.071 (2)	0.0562 (18)	0.0481 (17)	−0.0032 (15)	0.0288 (16)	0.0014 (14)
C19	0.064 (2)	0.0507 (18)	0.062 (2)	0.0030 (14)	0.0301 (16)	−0.0033 (14)
C20	0.0485 (17)	0.0427 (16)	0.0580 (19)	0.0012 (13)	0.0182 (15)	−0.0043 (14)
C21	0.0526 (18)	0.0419 (16)	0.0576 (19)	−0.0071 (13)	0.0222 (15)	−0.0049 (13)
C22	0.085 (2)	0.0462 (18)	0.076 (2)	−0.0164 (16)	0.038 (2)	−0.0129 (16)
C23	0.118 (3)	0.056 (2)	0.102 (3)	−0.025 (2)	0.067 (3)	−0.010 (2)
C24	0.137 (4)	0.052 (2)	0.124 (4)	−0.034 (2)	0.078 (3)	−0.014 (2)
C25	0.126 (3)	0.046 (2)	0.109 (3)	−0.021 (2)	0.061 (3)	−0.021 (2)
C26	0.071 (2)	0.0416 (17)	0.076 (2)	−0.0046 (14)	0.0355 (18)	−0.0061 (15)
C27	0.0584 (19)	0.0545 (18)	0.0450 (17)	−0.0018 (14)	0.0113 (15)	−0.0084 (14)
C28	0.0476 (17)	0.0537 (16)	0.0385 (16)	−0.0039 (13)	0.0089 (14)	−0.0003 (13)
C29	0.0502 (17)	0.0471 (16)	0.0515 (18)	0.0021 (13)	0.0122 (14)	0.0004 (13)
C30	0.067 (2)	0.067 (2)	0.068 (2)	0.0117 (17)	0.0033 (18)	−0.0148 (18)
C31	0.084 (3)	0.062 (2)	0.098 (3)	0.0080 (19)	0.024 (2)	−0.019 (2)
C32	0.061 (2)	0.064 (2)	0.096 (3)	0.0147 (17)	0.015 (2)	0.004 (2)
C33	0.059 (2)	0.069 (2)	0.066 (2)	0.0101 (17)	0.0093 (17)	0.0084 (18)
C34	0.0456 (17)	0.0530 (17)	0.0482 (17)	−0.0010 (14)	0.0102 (14)	0.0057 (14)
C35	0.120 (5)	0.140 (5)	0.141 (5)	−0.007 (4)	0.054 (4)	−0.030 (4)
C36	0.079 (3)	0.154 (5)	0.186 (6)	−0.009 (3)	0.054 (4)	−0.033 (4)
C37	0.193 (6)	0.135 (5)	0.122 (5)	−0.006 (4)	0.059 (5)	0.003 (4)
N1	0.0480 (14)	0.0466 (14)	0.0518 (14)	−0.0044 (10)	0.0176 (12)	0.0054 (11)
N2	0.0498 (14)	0.0475 (14)	0.0441 (14)	0.0013 (11)	0.0172 (11)	0.0011 (10)
N3	0.0591 (15)	0.0444 (13)	0.0510 (15)	−0.0113 (11)	0.0176 (12)	−0.0040 (11)
N4	0.126 (3)	0.0428 (16)	0.097 (2)	−0.0225 (16)	0.057 (2)	0.0000 (15)
N5	0.0382 (13)	0.0489 (14)	0.0597 (15)	−0.0036 (10)	0.0138 (12)	0.0055 (11)
N6	0.0340 (13)	0.0583 (15)	0.0665 (17)	−0.0058 (11)	0.0134 (12)	−0.0002 (13)
N7	0.0517 (14)	0.0411 (13)	0.0555 (15)	−0.0048 (11)	0.0219 (12)	−0.0065 (11)
N8	0.0808 (19)	0.0448 (14)	0.0768 (19)	−0.0018 (13)	0.0407 (16)	−0.0149 (13)
N9	0.0536 (15)	0.0523 (14)	0.0424 (14)	0.0042 (11)	0.0063 (11)	−0.0014 (11)
N10	0.0521 (15)	0.0625 (16)	0.0404 (14)	0.0001 (12)	0.0009 (12)	−0.0049 (12)
N11	0.0423 (14)	0.0449 (13)	0.0499 (15)	−0.0071 (10)	0.0152 (12)	0.0003 (11)
O1	0.0349 (11)	0.0757 (14)	0.0457 (12)	−0.0008 (9)	0.0107 (9)	0.0087 (10)
O2	0.0432 (11)	0.0752 (15)	0.0525 (13)	0.0030 (10)	0.0025 (10)	0.0073 (11)
O3	0.0608 (14)	0.0940 (17)	0.0617 (15)	−0.0026 (12)	0.0334 (12)	0.0064 (12)
N12	0.169 (9)	0.063 (6)	0.134 (12)	−0.034 (5)	0.107 (10)	−0.030 (6)
O4	0.319 (12)	0.123 (6)	0.252 (11)	0.008 (7)	0.214 (10)	−0.065 (6)
O5	0.243 (10)	0.101 (4)	0.126 (5)	−0.001 (5)	0.058 (6)	−0.003 (4)
O6	0.150 (7)	0.116 (6)	0.179 (8)	−0.010 (5)	0.077 (6)	−0.051 (5)
O7	0.128 (3)	0.170 (4)	0.132 (3)	−0.008 (3)	0.018 (2)	−0.053 (3)
O8	0.096 (3)	0.113 (3)	0.342 (7)	0.029 (2)	0.096 (3)	0.051 (4)
O9	0.299 (7)	0.161 (4)	0.176 (5)	−0.108 (4)	0.086 (5)	−0.012 (4)
O5'	0.037 (7)	0.111 (15)	0.19 (2)	−0.029 (8)	−0.048 (11)	0.074 (15)
O6'	0.157 (16)	0.079 (11)	0.068 (8)	−0.031 (10)	0.031 (10)	0.001 (7)
N12'	0.14 (3)	0.12 (3)	0.14 (4)	−0.03 (2)	0.05 (3)	0.00 (3)
O4'	0.13 (2)	0.090 (15)	0.130 (18)	0.016 (14)	0.034 (12)	−0.066 (12)

### Geometric parameters (Å, °)

**Table:**

Ca1—O1	2.440 (2)	C21—N7	1.396 (4)
Ca1—N9	2.470 (2)	C21—C26	1.404 (4)
Ca1—N5	2.477 (2)	C22—C23	1.377 (4)
Ca1—N3	2.521 (2)	C22—H22	0.93
Ca1—N7	2.543 (2)	C23—C24	1.396 (5)
Ca1—O2	2.641 (2)	C23—H23	0.93
Ca1—N1	2.647 (2)	C24—C25	1.349 (5)
Ca1—N2	2.649 (2)	C24—H24	0.93
C1—N1	1.474 (4)	C25—C26	1.383 (5)
C1—C2	1.484 (4)	C25—H25	0.93
C1—H1A	0.97	C26—N8	1.384 (4)
C1—H1B	0.97	C27—N2	1.462 (3)
C2—N3	1.328 (4)	C27—C28	1.496 (4)
C2—N4	1.331 (4)	C27—H27A	0.97
C3—N3	1.387 (4)	C27—H27B	0.97
C3—C8	1.389 (4)	C28—N9	1.315 (3)
C3—C4	1.394 (5)	C28—N10	1.326 (3)
C4—C5	1.380 (5)	C29—C34	1.376 (4)
C4—H4	0.93	C29—C30	1.382 (4)
C5—C6	1.394 (6)	C29—N9	1.407 (3)
C5—H5	0.93	C30—C31	1.368 (5)
C6—C7	1.348 (6)	C30—H30	0.93
C6—H6	0.93	C31—C32	1.379 (5)
C7—C8	1.385 (5)	C31—H31	0.93
C7—H7	0.93	C32—C33	1.357 (5)
C8—N4	1.375 (4)	C32—H32	0.93
C9—N1	1.462 (4)	C33—C34	1.370 (4)
C9—C10	1.488 (4)	C33—H33	0.93
C9—H9A	0.97	C34—N10	1.388 (4)
C9—H9B	0.97	C35—O7	1.377 (6)
C10—N5	1.324 (3)	C35—H35A	0.96
C10—N6	1.334 (3)	C35—H35B	0.96
C11—C16	1.386 (4)	C35—H35C	0.96
C11—N5	1.391 (3)	C36—O8	1.308 (5)
C11—C12	1.395 (4)	C36—H36A	0.96
C12—C13	1.362 (4)	C36—H36B	0.96
C12—H12	0.93	C36—H36C	0.96
C13—C14	1.381 (4)	C37—O9	1.328 (6)
C13—H13	0.93	C37—H37A	0.96
C14—C15	1.377 (5)	C37—H37B	0.96
C14—H14	0.93	C37—H37C	0.96
C15—C16	1.366 (4)	N4—H4A	0.86
C15—H15	0.93	N6—H6A	0.86
C16—N6	1.388 (4)	N8—H8	0.86
C17—C18	1.480 (4)	N10—H10	0.86
C17—N1	1.488 (4)	N11—O3	1.229 (3)
C17—H17A	0.97	N11—O2	1.244 (3)
C17—H17B	0.97	N11—O1	1.257 (3)
C18—N2	1.476 (4)	N12—O5	1.245 (8)
C18—H18A	0.97	N12—O6	1.275 (7)
C18—H18B	0.97	N12—O4	1.283 (8)
C19—N2	1.472 (4)	O7—H7A	0.82
C19—C20	1.490 (4)	O8—H8A	0.82
C19—H19A	0.97	O9—H9	0.82
C19—H19B	0.97	O5'—N12'	1.301 (10)
C20—N7	1.316 (3)	O6'—N12'	1.297 (10)
C20—N8	1.340 (4)	N12'—O4'	1.292 (10)
C21—C22	1.382 (4)		
			
O1—Ca1—N9	123.93 (8)	C21—C22—H22	120.4
O1—Ca1—N5	78.72 (7)	C22—C23—C24	119.6 (3)
N9—Ca1—N5	156.91 (8)	C22—C23—H23	120.2
O1—Ca1—N3	83.69 (8)	C24—C23—H23	120.2
N9—Ca1—N3	85.31 (8)	C25—C24—C23	123.0 (3)
N5—Ca1—N3	93.72 (8)	C25—C24—H24	118.5
O1—Ca1—N7	86.46 (8)	C23—C24—H24	118.5
N9—Ca1—N7	100.42 (8)	C24—C25—C26	117.1 (3)
N5—Ca1—N7	84.15 (8)	C24—C25—H25	121.4
N3—Ca1—N7	170.14 (8)	C26—C25—H25	121.4
O1—Ca1—O2	49.45 (6)	C25—C26—N8	133.2 (3)
N9—Ca1—O2	75.46 (7)	C25—C26—C21	121.8 (3)
N5—Ca1—O2	127.61 (7)	N8—C26—C21	105.0 (2)
N3—Ca1—O2	88.27 (7)	N2—C27—C28	109.7 (2)
N7—Ca1—O2	85.46 (7)	N2—C27—H27A	109.7
O1—Ca1—N1	131.28 (7)	C28—C27—H27A	109.7
N9—Ca1—N1	92.11 (8)	N2—C27—H27B	109.7
N5—Ca1—N1	66.68 (7)	C28—C27—H27B	109.7
N3—Ca1—N1	66.25 (8)	H27A—C27—H27B	108.2
N7—Ca1—N1	121.07 (8)	N9—C28—N10	113.3 (3)
O2—Ca1—N1	152.71 (7)	N9—C28—C27	123.7 (2)
O1—Ca1—N2	152.31 (7)	N10—C28—C27	123.0 (2)
N9—Ca1—N2	65.84 (7)	C34—C29—C30	120.0 (3)
N5—Ca1—N2	96.39 (8)	C34—C29—N9	109.3 (3)
N3—Ca1—N2	123.96 (8)	C30—C29—N9	130.7 (3)
N7—Ca1—N2	65.88 (7)	C31—C30—C29	117.5 (3)
O2—Ca1—N2	124.98 (7)	C31—C30—H30	121.2
N1—Ca1—N2	67.97 (7)	C29—C30—H30	121.2
N1—C1—C2	109.5 (2)	C30—C31—C32	121.5 (3)
N1—C1—H1A	109.8	C30—C31—H31	119.3
C2—C1—H1A	109.8	C32—C31—H31	119.3
N1—C1—H1B	109.8	C33—C32—C31	121.5 (3)
C2—C1—H1B	109.8	C33—C32—H32	119.2
H1A—C1—H1B	108.2	C31—C32—H32	119.2
N3—C2—N4	112.1 (3)	C32—C33—C34	117.1 (3)
N3—C2—C1	124.4 (3)	C32—C33—H33	121.4
N4—C2—C1	123.5 (3)	C34—C33—H33	121.4
N3—C3—C8	109.3 (3)	C33—C34—C29	122.4 (3)
N3—C3—C4	131.7 (3)	C33—C34—N10	132.3 (3)
C8—C3—C4	119.0 (3)	C29—C34—N10	105.3 (2)
C5—C4—C3	118.6 (4)	O7—C35—H35A	109.5
C5—C4—H4	120.7	O7—C35—H35B	109.5
C3—C4—H4	120.7	H35A—C35—H35B	109.5
C4—C5—C6	120.5 (4)	O7—C35—H35C	109.5
C4—C5—H5	119.8	H35A—C35—H35C	109.5
C6—C5—H5	119.8	H35B—C35—H35C	109.5
C7—C6—C5	122.0 (4)	O8—C36—H36A	109.5
C7—C6—H6	119.0	O8—C36—H36B	109.5
C5—C6—H6	119.0	H36A—C36—H36B	109.5
C6—C7—C8	117.6 (4)	O8—C36—H36C	109.5
C6—C7—H7	121.2	H36A—C36—H36C	109.5
C8—C7—H7	121.2	H36B—C36—H36C	109.5
N4—C8—C7	132.5 (4)	O9—C37—H37A	109.5
N4—C8—C3	105.1 (3)	O9—C37—H37B	109.5
C7—C8—C3	122.4 (4)	H37A—C37—H37B	109.5
N1—C9—C10	110.9 (2)	O9—C37—H37C	109.5
N1—C9—H9A	109.5	H37A—C37—H37C	109.5
C10—C9—H9A	109.5	H37B—C37—H37C	109.5
N1—C9—H9B	109.5	C9—N1—C1	110.0 (2)
C10—C9—H9B	109.5	C9—N1—C17	109.4 (2)
H9A—C9—H9B	108.1	C1—N1—C17	111.9 (2)
N5—C10—N6	112.7 (3)	C9—N1—Ca1	110.18 (16)
N5—C10—C9	124.1 (2)	C1—N1—Ca1	104.11 (17)
N6—C10—C9	123.0 (2)	C17—N1—Ca1	111.23 (16)
C16—C11—N5	109.7 (2)	C27—N2—C19	109.9 (2)
C16—C11—C12	119.7 (3)	C27—N2—C18	109.4 (2)
N5—C11—C12	130.6 (3)	C19—N2—C18	111.4 (2)
C13—C12—C11	117.7 (3)	C27—N2—Ca1	108.73 (16)
C13—C12—H12	121.2	C19—N2—Ca1	106.49 (17)
C11—C12—H12	121.2	C18—N2—Ca1	110.85 (17)
C12—C13—C14	122.1 (3)	C2—N3—C3	105.2 (2)
C12—C13—H13	119.0	C2—N3—Ca1	112.57 (19)
C14—C13—H13	119.0	C3—N3—Ca1	141.8 (2)
C15—C14—C13	120.7 (3)	C2—N4—C8	108.3 (3)
C15—C14—H14	119.7	C2—N4—H4A	125.8
C13—C14—H14	119.7	C8—N4—H4A	125.8
C16—C15—C14	117.5 (3)	C10—N5—C11	104.9 (2)
C16—C15—H15	121.2	C10—N5—Ca1	117.35 (18)
C14—C15—H15	121.2	C11—N5—Ca1	137.37 (18)
C15—C16—C11	122.3 (3)	C10—N6—C16	107.8 (2)
C15—C16—N6	132.8 (3)	C10—N6—H6A	126.1
C11—C16—N6	104.9 (2)	C16—N6—H6A	126.1
C18—C17—N1	113.1 (2)	C20—N7—C21	105.6 (2)
C18—C17—H17A	109.0	C20—N7—Ca1	114.01 (18)
N1—C17—H17A	109.0	C21—N7—Ca1	140.40 (19)
C18—C17—H17B	109.0	C20—N8—C26	107.9 (2)
N1—C17—H17B	109.0	C20—N8—H8	126.0
H17A—C17—H17B	107.8	C26—N8—H8	126.0
N2—C18—C17	113.5 (2)	C28—N9—C29	104.5 (2)
N2—C18—H18A	108.9	C28—N9—Ca1	117.15 (19)
C17—C18—H18A	108.9	C29—N9—Ca1	137.61 (18)
N2—C18—H18B	108.9	C28—N10—C34	107.7 (2)
C17—C18—H18B	108.9	C28—N10—H10	126.2
H18A—C18—H18B	107.7	C34—N10—H10	126.2
N2—C19—C20	109.2 (2)	O3—N11—O2	122.4 (2)
N2—C19—H19A	109.8	O3—N11—O1	120.5 (2)
C20—C19—H19A	109.8	O2—N11—O1	117.1 (2)
N2—C19—H19B	109.8	N11—O1—Ca1	101.47 (15)
C20—C19—H19B	109.8	N11—O2—Ca1	92.01 (15)
H19A—C19—H19B	108.3	O5—N12—O6	122.0 (7)
N7—C20—N8	112.7 (3)	O5—N12—O4	121.1 (7)
N7—C20—C19	124.4 (3)	O6—N12—O4	116.8 (8)
N8—C20—C19	122.8 (3)	C35—O7—H7A	109.5
C22—C21—N7	131.8 (3)	C36—O8—H8A	109.5
C22—C21—C26	119.4 (3)	C37—O9—H9	109.5
N7—C21—C26	108.8 (3)	O4'—N12'—O6'	118.1 (10)
C23—C22—C21	119.1 (3)	O4'—N12'—O5'	117.4 (10)
C23—C22—H22	120.4	O6'—N12'—O5'	116.7 (9)
			
N1—C1—C2—N3	32.3 (4)	C1—C2—N3—Ca1	6.9 (4)
N1—C1—C2—N4	−146.5 (3)	C8—C3—N3—C2	0.4 (4)
N3—C3—C4—C5	−179.8 (4)	C4—C3—N3—C2	178.9 (4)
C8—C3—C4—C5	−1.5 (6)	C8—C3—N3—Ca1	172.0 (3)
C3—C4—C5—C6	1.4 (7)	C4—C3—N3—Ca1	−9.6 (6)
C4—C5—C6—C7	−0.6 (9)	O1—Ca1—N3—C2	−165.3 (2)
C5—C6—C7—C8	−0.1 (10)	N9—Ca1—N3—C2	69.7 (2)
C6—C7—C8—N4	−179.7 (5)	N5—Ca1—N3—C2	−87.1 (2)
C6—C7—C8—C3	0.1 (9)	O2—Ca1—N3—C2	145.3 (2)
N3—C3—C8—N4	−0.8 (4)	N1—Ca1—N3—C2	−24.65 (19)
C4—C3—C8—N4	−179.4 (3)	N2—Ca1—N3—C2	13.1 (2)
N3—C3—C8—C7	179.4 (4)	O1—Ca1—N3—C3	23.5 (3)
C4—C3—C8—C7	0.7 (7)	N9—Ca1—N3—C3	−101.5 (3)
N1—C9—C10—N5	26.2 (4)	N5—Ca1—N3—C3	101.7 (3)
N1—C9—C10—N6	−159.9 (3)	O2—Ca1—N3—C3	−25.9 (3)
C16—C11—C12—C13	0.6 (4)	N1—Ca1—N3—C3	164.2 (3)
N5—C11—C12—C13	177.2 (3)	N2—Ca1—N3—C3	−158.1 (3)
C11—C12—C13—C14	0.2 (5)	N3—C2—N4—C8	−0.6 (4)
C12—C13—C14—C15	−0.1 (5)	C1—C2—N4—C8	178.3 (3)
C13—C14—C15—C16	−0.9 (5)	C7—C8—N4—C2	−179.3 (5)
C14—C15—C16—C11	1.8 (4)	C3—C8—N4—C2	0.8 (4)
C14—C15—C16—N6	−177.8 (3)	N6—C10—N5—C11	−0.2 (3)
N5—C11—C16—C15	−178.9 (3)	C9—C10—N5—C11	174.3 (3)
C12—C11—C16—C15	−1.7 (4)	N6—C10—N5—Ca1	−174.70 (18)
N5—C11—C16—N6	0.7 (3)	C9—C10—N5—Ca1	−0.2 (4)
C12—C11—C16—N6	178.0 (3)	C16—C11—N5—C10	−0.4 (3)
N1—C17—C18—N2	57.6 (3)	C12—C11—N5—C10	−177.2 (3)
N2—C19—C20—N7	33.5 (4)	C16—C11—N5—Ca1	172.5 (2)
N2—C19—C20—N8	−144.3 (3)	C12—C11—N5—Ca1	−4.4 (5)
N7—C21—C22—C23	−178.7 (3)	O1—Ca1—N5—C10	130.8 (2)
C26—C21—C22—C23	−0.1 (5)	N9—Ca1—N5—C10	−38.7 (3)
C21—C22—C23—C24	0.7 (6)	N3—Ca1—N5—C10	48.0 (2)
C22—C23—C24—C25	−0.4 (7)	N7—Ca1—N5—C10	−141.6 (2)
C23—C24—C25—C26	−0.5 (7)	O2—Ca1—N5—C10	138.70 (19)
C24—C25—C26—N8	178.7 (4)	N1—Ca1—N5—C10	−14.1 (2)
C24—C25—C26—C21	1.1 (6)	N2—Ca1—N5—C10	−76.8 (2)
C22—C21—C26—C25	−0.8 (5)	O1—Ca1—N5—C11	−41.4 (3)
N7—C21—C26—C25	178.1 (3)	N9—Ca1—N5—C11	149.1 (2)
C22—C21—C26—N8	−179.0 (3)	N3—Ca1—N5—C11	−124.2 (3)
N7—C21—C26—N8	0.0 (3)	N7—Ca1—N5—C11	46.2 (3)
N2—C27—C28—N9	25.6 (4)	O2—Ca1—N5—C11	−33.5 (3)
N2—C27—C28—N10	−156.4 (3)	N1—Ca1—N5—C11	173.7 (3)
C34—C29—C30—C31	1.1 (5)	N2—Ca1—N5—C11	111.0 (3)
N9—C29—C30—C31	−179.7 (3)	N5—C10—N6—C16	0.6 (3)
C29—C30—C31—C32	0.5 (6)	C9—C10—N6—C16	−173.9 (3)
C30—C31—C32—C33	−1.5 (6)	C15—C16—N6—C10	178.8 (3)
C31—C32—C33—C34	0.7 (6)	C11—C16—N6—C10	−0.8 (3)
C32—C33—C34—C29	0.9 (5)	N8—C20—N7—C21	−1.9 (3)
C32—C33—C34—N10	179.9 (3)	C19—C20—N7—C21	−179.9 (3)
C30—C29—C34—C33	−1.9 (5)	N8—C20—N7—Ca1	179.92 (19)
N9—C29—C34—C33	178.8 (3)	C19—C20—N7—Ca1	1.9 (3)
C30—C29—C34—N10	178.9 (3)	C22—C21—N7—C20	179.9 (3)
N9—C29—C34—N10	−0.5 (3)	C26—C21—N7—C20	1.1 (3)
C10—C9—N1—C1	−149.5 (3)	C22—C21—N7—Ca1	−2.7 (5)
C10—C9—N1—C17	87.3 (3)	C26—C21—N7—Ca1	178.5 (2)
C10—C9—N1—Ca1	−35.3 (3)	O1—Ca1—N7—C20	158.32 (19)
C2—C1—N1—C9	68.8 (3)	N9—Ca1—N7—C20	−77.8 (2)
C2—C1—N1—C17	−169.4 (2)	N5—Ca1—N7—C20	79.3 (2)
C2—C1—N1—Ca1	−49.2 (3)	O2—Ca1—N7—C20	−152.1 (2)
C18—C17—N1—C9	−162.2 (2)	N1—Ca1—N7—C20	21.1 (2)
C18—C17—N1—C1	75.7 (3)	N2—Ca1—N7—C20	−20.38 (18)
C18—C17—N1—Ca1	−40.2 (3)	O1—Ca1—N7—C21	−18.9 (3)
O1—Ca1—N1—C9	−22.1 (2)	N9—Ca1—N7—C21	104.9 (3)
N9—Ca1—N1—C9	−162.98 (19)	N5—Ca1—N7—C21	−98.0 (3)
N5—Ca1—N1—C9	26.43 (18)	O2—Ca1—N7—C21	30.6 (3)
N3—Ca1—N1—C9	−79.05 (19)	N1—Ca1—N7—C21	−156.2 (3)
N7—Ca1—N1—C9	93.51 (19)	N2—Ca1—N7—C21	162.4 (3)
O2—Ca1—N1—C9	−101.5 (2)	N7—C20—N8—C26	1.9 (3)
N2—Ca1—N1—C9	134.2 (2)	C19—C20—N8—C26	−180.0 (3)
O1—Ca1—N1—C1	95.72 (18)	C25—C26—N8—C20	−178.9 (4)
N9—Ca1—N1—C1	−45.13 (18)	C21—C26—N8—C20	−1.1 (3)
N5—Ca1—N1—C1	144.28 (19)	N10—C28—N9—C29	−0.3 (3)
N3—Ca1—N1—C1	38.81 (17)	C27—C28—N9—C29	177.8 (3)
N7—Ca1—N1—C1	−148.63 (17)	N10—C28—N9—Ca1	−172.16 (19)
O2—Ca1—N1—C1	16.4 (3)	C27—C28—N9—Ca1	6.0 (4)
N2—Ca1—N1—C1	−107.96 (18)	C34—C29—N9—C28	0.5 (3)
O1—Ca1—N1—C17	−143.63 (17)	C30—C29—N9—C28	−178.7 (3)
N9—Ca1—N1—C17	75.53 (18)	C34—C29—N9—Ca1	169.7 (2)
N5—Ca1—N1—C17	−95.06 (19)	C30—C29—N9—Ca1	−9.6 (5)
N3—Ca1—N1—C17	159.5 (2)	O1—Ca1—N9—C28	130.0 (2)
N7—Ca1—N1—C17	−28.0 (2)	N5—Ca1—N9—C28	−62.4 (3)
O2—Ca1—N1—C17	137.05 (19)	N3—Ca1—N9—C28	−150.9 (2)
N2—Ca1—N1—C17	12.70 (17)	N7—Ca1—N9—C28	37.2 (2)
C28—C27—N2—C19	−156.1 (2)	O2—Ca1—N9—C28	119.7 (2)
C28—C27—N2—C18	81.3 (3)	N1—Ca1—N9—C28	−84.9 (2)
C28—C27—N2—Ca1	−39.9 (3)	N2—Ca1—N9—C28	−20.3 (2)
C20—C19—N2—C27	70.5 (3)	O1—Ca1—N9—C29	−38.3 (3)
C20—C19—N2—C18	−168.0 (2)	N5—Ca1—N9—C29	129.4 (3)
C20—C19—N2—Ca1	−47.1 (3)	N3—Ca1—N9—C29	40.9 (3)
C17—C18—N2—C27	−162.4 (2)	N7—Ca1—N9—C29	−131.0 (3)
C17—C18—N2—C19	75.9 (3)	O2—Ca1—N9—C29	−48.5 (3)
C17—C18—N2—Ca1	−42.5 (3)	N1—Ca1—N9—C29	106.8 (3)
O1—Ca1—N2—C27	−85.5 (2)	N2—Ca1—N9—C29	171.5 (3)
N9—Ca1—N2—C27	32.01 (17)	N9—C28—N10—C34	0.0 (3)
N5—Ca1—N2—C27	−163.35 (17)	C27—C28—N10—C34	−178.1 (3)
N3—Ca1—N2—C27	97.79 (18)	C33—C34—N10—C28	−178.9 (3)
N7—Ca1—N2—C27	−82.70 (17)	C29—C34—N10—C28	0.3 (3)
O2—Ca1—N2—C27	−17.5 (2)	O3—N11—O1—Ca1	−178.7 (2)
N1—Ca1—N2—C27	135.01 (18)	O2—N11—O1—Ca1	−0.3 (2)
O1—Ca1—N2—C19	32.9 (2)	N9—Ca1—O1—N11	−12.96 (18)
N9—Ca1—N2—C19	150.38 (19)	N5—Ca1—O1—N11	171.96 (16)
N5—Ca1—N2—C19	−44.97 (17)	N3—Ca1—O1—N11	−92.96 (16)
N3—Ca1—N2—C19	−143.84 (17)	N7—Ca1—O1—N11	87.23 (16)
N7—Ca1—N2—C19	35.67 (17)	O2—Ca1—O1—N11	0.18 (13)
O2—Ca1—N2—C19	100.90 (17)	N1—Ca1—O1—N11	−143.45 (14)
N1—Ca1—N2—C19	−106.62 (18)	N2—Ca1—O1—N11	89.8 (2)
O1—Ca1—N2—C18	154.23 (18)	O3—N11—O2—Ca1	178.6 (2)
N9—Ca1—N2—C18	−88.26 (18)	O1—N11—O2—Ca1	0.3 (2)
N5—Ca1—N2—C18	76.38 (18)	O1—Ca1—O2—N11	−0.18 (13)
N3—Ca1—N2—C18	−22.5 (2)	N9—Ca1—O2—N11	168.59 (16)
N7—Ca1—N2—C18	157.03 (19)	N5—Ca1—O2—N11	−10.37 (18)
O2—Ca1—N2—C18	−137.75 (17)	N3—Ca1—O2—N11	82.99 (15)
N1—Ca1—N2—C18	14.73 (17)	N7—Ca1—O2—N11	−89.40 (15)
N4—C2—N3—C3	0.1 (4)	N1—Ca1—O2—N11	103.43 (19)
C1—C2—N3—C3	−178.8 (3)	N2—Ca1—O2—N11	−145.63 (14)
N4—C2—N3—Ca1	−174.3 (2)		

### Hydrogen-bond geometry (Å, °)

**Table:**

*D*—H···*A*	*D*—H	H···*A*	*D*···*A*	*D*—H···*A*
N4—H4A···O5^i^	0.86	2.46	3.208 (8)	145
N4—H4A···O6^i^	0.86	2.09	2.888 (10)	154
N6—H6A···O2^ii^	0.86	2.26	3.099 (3)	165
N6—H6A···O3^ii^	0.86	2.45	3.118 (3)	135
N10—H10···O1^iii^	0.86	1.94	2.789 (3)	172
O7—H7A···O4^iv^	0.82	2.39	3.109 (11)	147
N8—H8···O4	0.86	1.92	2.730 (7)	157
O8—H8A···O9	0.82	1.86	2.655 (6)	164
O9—H9···O5	0.82	2.14	2.929 (9)	160
C9—H9B···O7	0.97	2.39	3.274 (5)	151
C17—H17A···O8	0.97	2.33	3.290 (5)	168
C19—H19B···O8	0.97	2.45	3.378 (5)	160

Symmetry codes: (i) −*x*+3/2, *y*−1/2, −*z*+3/2; (ii) *x*−1, *y*, *z*; (iii) *x*+1/2, −*y*+1/2, *z*+1/2; (iv) *x*−1/2, −*y*+1/2, *z*−1/2.
